# Environmental drivers of fungal community structure: a comparative high-throughput sequencing study of two geothermally distinct hot springs in Hefei, China

**DOI:** 10.3389/fmicb.2025.1477445

**Published:** 2025-07-17

**Authors:** Feng-Qin Zhang

**Affiliations:** College of Biological and Environmental Engineering, Chaohu University, Chaohu, Anhui, China

**Keywords:** fungal community structure, environmental drivers, high-throughput sequencing, Bantang hot spring, Tangchi hot spring

## Abstract

**Introduction:**

There are abundant hot spring resources in Hefei. This is the first study to analyze and compare the fungal diversity of Tangchi and Bantang hot springs in Hefei.

**Methods:**

This study represents the comprehensive analysis of fungal diversity in two geothermally distinct hot springs (Tangchi and Bantang) in Hefei, China, using Illumina MiSeq PE300 sequencing of the ITS region.

**Results:**

Despite their spatial proximity, Tangchi (63.9°C, pH 8.2) and Bantang (45.8°C, pH 6.5) showed stark contrasts in fungal community composition. A total of 934 OTUs were identified, with Tangchi exhibiting greater α-diversity and only 18.6% OTU overlap. Both systems were dominated by Ascomycota and Basidiomycota, yet genus-level divergences were substantial: Tangchi favored *Rhizophydium, Aureobasidium*, and *Rhodotorula*, while Bantang harbored *Coniosporium, Trichomerium*, and *Aureobasidium*.

**Discussion:**

Comparative analysis with freshwater ecosystems revealed unique thermoadaptive strategies and reduced overlap with non-thermal fungal assemblages. These findings emphasize temperature and pH as critical drivers of fungal niche partitioning in geothermal systems and underscore the untapped biotechnological potential of thermophilic fungi.

## 1 Introduction

Freshwater ecosystems are among the most vital on earth, playing key roles in the global water cycle, matter cycling, and energy flow. Freshwater ecosystems support a diverse array of organisms, ranging from microorganisms such as bacteria, fungi, and protists to macroscopic organisms like plankton, aquatic plants, and animals. Fungi are key yet understudied components of aquatic ecosystems. These organisms play essential roles in nutrient cycling, organic matter decomposition, and symbiotic interactions. To date, over 3,000 species have been described; however, molecular studies highlight the substantial undiscovered diversity, particularly in tropical systems (Shearer et al., 2007).

Geothermal springs represent unique ecosystems characterized by extreme physicochemical conditions (e.g., elevated temperatures, variable pH, and mineral-rich waters), which influence microbial community structures and evolutionary adaptations (Power et al., 2018). Fungal communities in these environments have attracted significant research attention due to their thermotolerance, metabolic versatility, and potential applications in biotechnology and ecological restoration (Mysen, 2016; Ozdemir and Uzel, 2020; Arenas et al., 2022). However, compared to bacterial and archaeal diversity, fungal assemblages in geothermal systems, particularly in East Asian hot springs, are poorly understood. This gap in knowledge hinders our understanding of fungal ecological roles in extreme habitats and their responses to environmental factors.

The special geological conditions in Hefei, China, have endowed the region with abundant geothermal resources, and its hot springs has a long historical legacy. Currently, there are six known hot springs in Hefei. Among these, only the Bantang and Tangchi hot springs have been extensively developed for activities such as bathing, recuperation, and tourism. The remaining hot springs remain protected.

In an earlier study, the bacterial communities of Bantang and Tangchi hot springs were compared and analyzed using Illumina MiSeq PE300 (Zhang et al., 2023). The study employs Illumina MiSeq PE300 to classify and sequence the fungi present in the two hot springs. The primary aim is to conduct a comprehensive analysis and comparison of the fungal community diversity in both hot springs using high-throughput sequencing technology. This represents the first detailed examination of the fungal communities in these two hot springs, aiming to bridge gaps and provide theoretical support for the utilization of their fungal resources.

## 2 Materials and methods

### 2.1 Sampling

During August 2022, water samples were collected from two distinct geothermal springs in Hefei, China: Tangchi hot spring (117.54°E, 31.38°N) located in Lujiang district and Bantang hot spring (117.54°E, 31.38°N) in Chaohu district. Tangchi and Bantang hot springs are engineered as an artesian tube and an artesian well, respectively.

At each sampling site, triplicate 5 L water samples were aseptically collected into sterile containers. Immediately following collection, on-site measurements of temperature and pH were recorded using a multi-parameter water quality analyzer (YSI EXO2) to ensure real-time data accuracy and minimize post-sampling variability.

In the laboratory, triplicate samples from each location were homogenized into a composite sample. One liter of the composite sample was stored at 4°C for chemical analysis. The remaining water samples were then filtered through a 0.22 μm sterile membrane to concentrate microbial biomass. The resulting filter membranes were subsequently stored at −80°C until further analysis.

### 2.2 Physicochemical determinations

Chemical analysis of metal ions was performed using inductively coupled plasma optical emission spectrometry (ICP-OES). Total nitrogen was determined by an automatic total nitrogen detection method. Anions were measured via ion chromatography. Organic carbon was analyzed using the TOC (Total Organic Carbon) analysis method.

### 2.3 Fungal ITS high-throughput sequencing

Total genomic DNA was extracted from two hot spring samples using the E.Z.N.A™ Mag-Bind Soil DNA Kit (OMEGA). The integrity and concentration of DNA samples were assessed using agarose gel electrophoresis and Qubit 4.0 (Thermo), respectively.

The internal transcriptional spacer ITS region was used as the target sequence and amplified using universal primers ITS1F (CTTGGTCATTTAGAGGAAGTAA) (Gardes and Bruns, 1993)/ITS2R (GCTGCGTTCTTCATCGATGC) (White et al., 1990). The PCR amplification products were purified and homogenized, and a small fragment sequencing library was constructed using the Paired-End method. The constructed library underwent library quality inspection, and then the qualified library was sequenced three times on the Illumina MiSeq PE300 platform using the MiSeq Reagent Kit v3 (600 cycles, Illumina).

Following the Barcode and amplification primer sequences, the data for each sample were separated from the raw sequencing data. The Barcode and primer sequences were then removed, and the reads for each sample were spliced together (Zhu et al., 2022). The resulting raw tags were filtered to remove low-quality tags and chimeras that did not meet the required length, generating clean tags (Lu et al., 2020).

### 2.4 Bioinformatics analysis

High-quality ITS sequences were merged and clustered into Operational Taxonomic Units (OTUs) at a 97% similarity threshold using Usearch (version 11.0.667). Chimeric sequences and singleton OTUs were systematically removed to minimize artifacts, as previously described. Representative OTU sequences were annotated against the UNITE database (v9.0) using the Blast algorithm, identical to our prior methodology, to maintain taxonomic consistency across both Tangchi and Bantang hot spring datasets. Four α-diversity indices (Ace, Chao1, Shannon, and Simpson) were calculated for each sample using Mothur (version 3.8.31). This consistency enabled direct comparison of microbial diversity patterns between the two hot springs. Statistical analyses for this study were performed using SPSS software (version 16.0).

### 2.5 Data accession

ITS gene sequencing data were accepted at Sequence Read Archive (SRA) under the accession Nos.SRR20831103 and SRR20831104. Fungal OTU sequences were accepted at GenBank under the accession Nos. PV773522 - PV773934 and PV746814-PV747184.

## 3 Results

### 3.1 Water sample parameter of the two hot springs

The temperature and pH of water samples from Tangchi and Bantang hot springs were measured on-site. Two hot springs showed differences in physical and chemical properties such as temperature, pH, calcium, magnesium, bicarbonate ions, sulfate ions, total organic carbon, and dissolved organic carbon. Physical and chemical properties such as iron, zinc, and phosphate ions were not detected (the concentrations were too low) (Table 1). The temperature and pH of water samples from Tangchi hot spring were 63.9°C and 8.2, respectively, indicating that Tangchi hot spring is alkaline. In this study, for Bantang hot spring, the on-site measurement results showed a temperature of 45.8°Cand a pH of 6.5. This indicates that the Tangchi hot spring has a higher temperature and is alkaline, whereas the Bantang hot spring is cooler and slightly acidic.

**Table 1:** Physicochemical parameters for Tangchi and Bantang hot springs.

**Sample**	**Tangchi**	**Bantang**
Temperature (°C)	63.9	45.8
pH	8.2	6.5
Mn (mg/L)	0.013	0.011
Ca (mg/L)	41.9	393
Mg (mg/L)	0.227	83.3
Total nitrogen (mg/L)	0.323	0.301
Cl-(mg/L)	49.2	4.76
${\text{NO}}_{3}^{2-}$ (in N, mg/L)	0.297	0.252
${\text{SO}}_{4}^{2-}$ (mg/L)	686	1.27 × 10^3^
Total organic carbon (mg/L)	6.8	21.5
Dissolved organic carbon (mg/L)	5.2	20.7

### 3.2 Sequencing results of hot spring samples

A total of 104,626 reads were obtained from the ITS gene sequence libraries of two hot springs in Hefei, and 104,120 valid tags were extracted after merging. The Tangchi hot spring yielded 56,052 reads and 56,039 valid tags, while the Bantang hot spring produced 48,574 reads and 48,081 valid tags.

**Figure 1:**
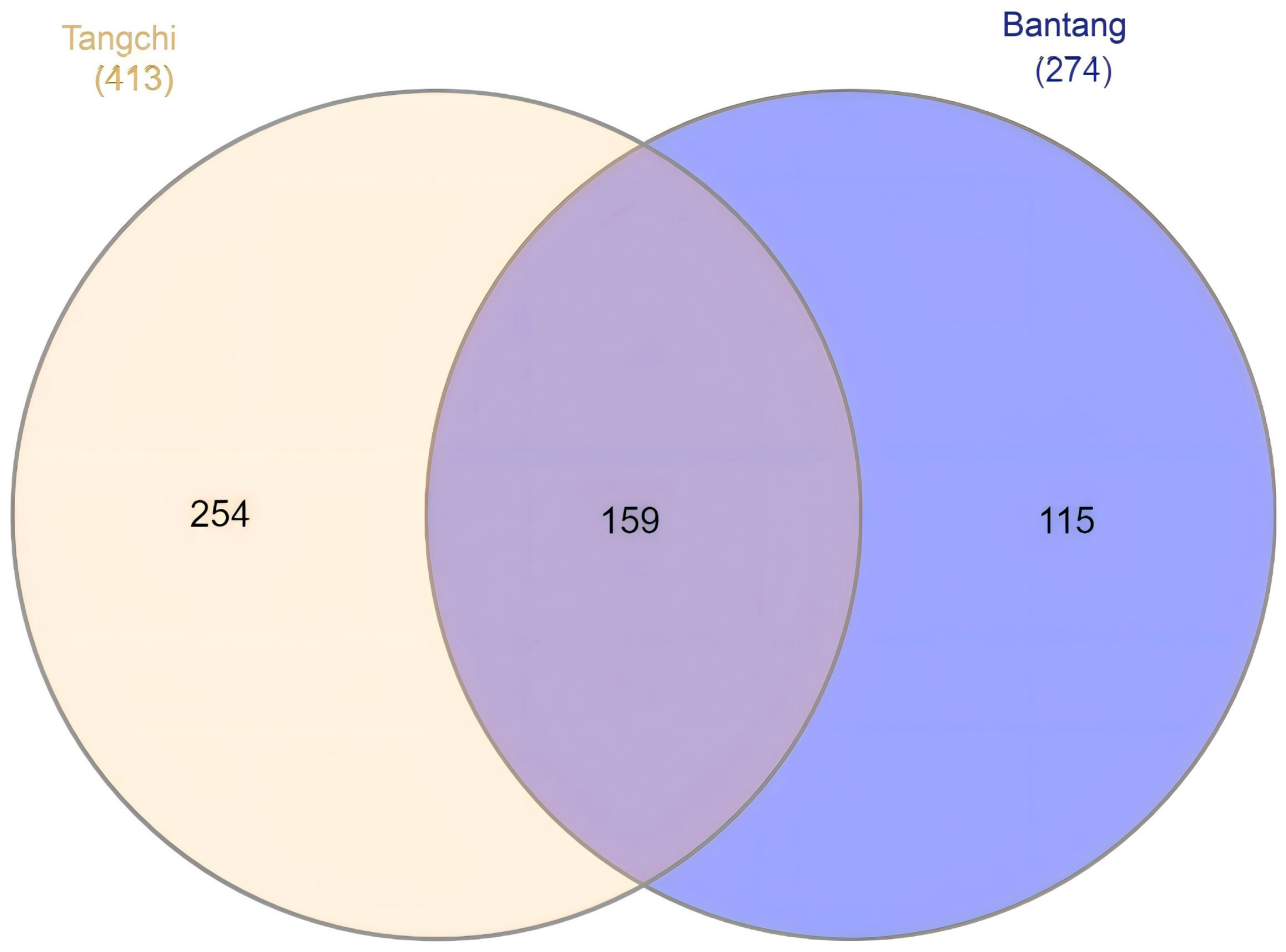
OTU numbers of fungal communities from two hot springs.

After splitting and removing redundancy, the sequences from the hot spring samples were clustered into OTUs under a similarity of 97%. In this study, a total of 687 OTUs were obtained from both hot springs (Figure 1), and the shared OTUs account for 23.1%. Bantang hot spring exhibited higher community overlap (58.0% of its OTUs shared), while Tangchi hot spring showed lower overlap (38.5% shared OTUs).

### 3.3 Composition of fungal community

The representative sequences of OTUs from the two hot springs were aligned. The results provided comprehensive information about the fungal communities in these hot springs. A total of 18 phyla, 57 classes, 126 orders, 265 families, 411 genera and 552 species were identified across both hot springs.

**Figure 2:**
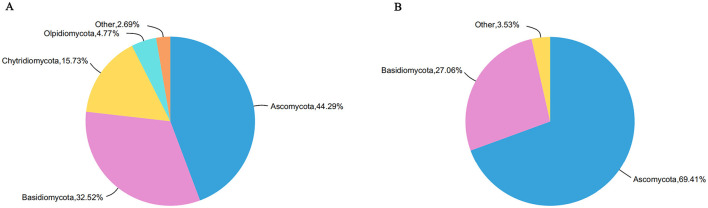
Phylum level distribution of fungal community in two hot springs: **(A)** Tangchi; **(B)** Bantang.

At the phylum taxonomic level, fungi with relative abundance <1% and unclassified fungi were attributed to others. The fungi communities in both the Tangchi and Bantang hot springs were primarily classified into 4 phyla (Figure 2). In the Tangchi hot spring, the predominant fungal phyla were Ascomycota (44.29%), Basidiomycota (32.52%), Chytridiomycota (15.73%), and Olpidiomycota (4.77%). In contrast, the Bantang hot spring had a different fungal phylum composition, with Ascomycota being the most abundant (69.41%), followed by Basidiomycota (27.06%).

**Figure 3:**
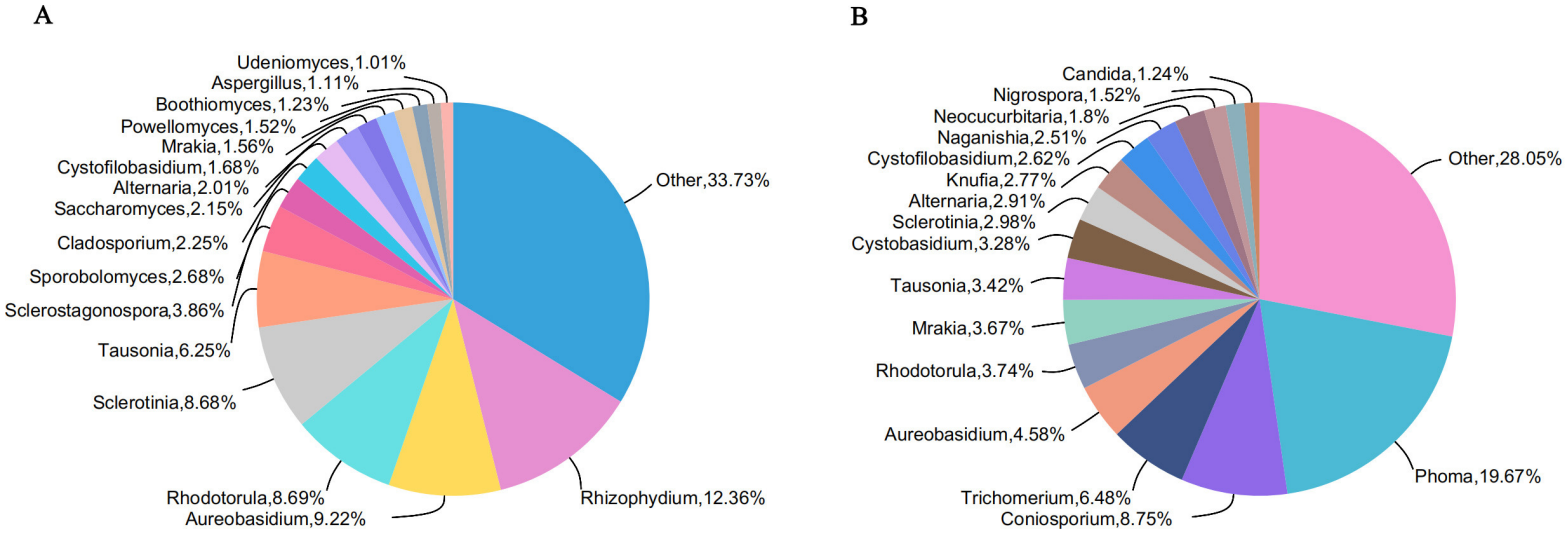
Genus level distribution of fungal community in two hot springs: **(A)** Tangchi; **(B)** Bantang.

In this study, two unclassified genera were grouped into the “other” category. The fungal communities in Tangchi hot spring were classified into 16 genera (Figure 3), with the most abundant being *Rhizophydium* (12.36%), *Aureobasidium* (9.22%), *Rhodotorula* (8.69%), *Sclerotinia* (8.68%), *Tausonia* (6.25%), *Sclerostagonospora* (3.86%), *Sporobolomyces* (2.68%), *Cladosporium* (2.25%), *Saccharomyces* (2.15%), *Alternaria* (2.01%), and genera with abundance >1% and <2%. In contrast, the fungal communities in Bantang hot spring primarily belonged to 16 genera, including *Phoma* (19.67%), *Coniosporium* (8.75%), *Trichomerium* (6.47%), *Aureobasidium* (4.58%), *Rhodotorula* (3.74%), *Mrakia* (3.67%), *Tausonia* (3.42%), *Cystobasidium* (3.28%), *Sclerotinia* (2.98%), *Alternaria* (2.91%), *Knufia* (2.77%), *Cystofilobasidium* (2.51%), and genera with abundance >1% and <2%.

The presence of unknown fungi in both hot springs (Tangchi 5.13%, Bantang 9.52%) suggests that the fungal genera in these environments possess a certain degree of novelty. Notably, *Rhizophydium, Sclerostagonospora, Sporobolomyces, Cladosporium, Saccharomyces, Powellomyces, Boothiomyces, Aspergillus*, and *Udeniomyces* were detected in Tangchi hot spring but not in Bantang hot spring. Conversely, *Phoma, Coniosporium, Trichomerium, Cystobasidium, Knufia, Naganishia, Neocucurbitaria, Nigrospora*, and *Candida* were discovered in Bantang hot spring but not in Tangchi hot spring. These findings indicate significant differences in the fungal genera present in the two hot springs.

### 3.4 Fungal alpha diversity analysis

Alpha diversity metrics provide insights into the richness, diversity and evenness of microbial community. In this study, we calculated alpha diversity indices and performed single-factor analysis of variance for fungal communities in the two hot springs (Table 2). The results indicated that the Shannon, Chao1, and ACE indices were higher for the Tangchi hot spring. Conversely, the Simpson index was lower for the Bantang hot spring. Notably, while there was no significant difference in the Shannon index between the two hot springs, other indices exhibited significant differences. These findings suggest that the fungal diversity in Tangchi hot spring was greater. Additionally, the coverage indices for both hot springs were close to 1, reflecting that the sequencing data accurately represented the actual conditions of the water samples.

**Table 2:** Fungal alpha diversity indexes of the two hot springs.

**Sample**	**Shannon**	**Chao**	**Ace**	**Simpson**	**Shannoneven**	**Coverage**
Tangchi	4.13 ± 0.12^a^	413.00 ± 22.23^a^	413.00 ± 23.75^a^	0.042 ± 0.001^a^	0.686 ± 0.009	1.0000 ± 0.0001
Bantang	3.96 ± 0.15^a^	274.00 ± 18.24^b^	274.00 ± 19.59^b^	0.058 ± 0.001^b^	0.706 ± 0.018	1.0000 ± 0.0001

The value is mean ± standard deviation; The same lowercase letter and different lowercase letters in the same column indicate no significant difference (*P* < 0.05) and significant differences (*P* < 0.05), respectively.

## 4 Discussion

Hot spring ecosystems, characterized by high temperatures and unique chemical environments, serve as ideal sites for studying microbial adaptation and diversity. The fungal communities in Tangchi hot spring (63.9°C, pH 8.2) and Bantang hot spring (45.8°C, pH 6.5) exhibit significant differences, which may be related to their respective environmental conditions.

With a relatively lower content of calcium, magnesium, and a moderate level of total organic carbon (6.8 mg/L) in Tangchi hot spring, the dominant phyla such as Ascomycota, Basidiomycota, and Chytridiomycota play crucial roles in organic matter decomposition. Chytridiomycota was detected in Tangchi hot spring, accounting for 15.73%, but was absent in Bantang hot spring. Chytridiomycota are rarely reported in other global thermal springs, with only trace amounts (<0.1%) documented in five hot springs at Rehai, Tengchong, Yunnan (Liu et al., 2017). Interestingly, it has been observed that Chytridiomycota constitute a dominant phylum in groundwater springs within the volcanic zone of Iceland (Wurzbacher et al., 2020) and assume dual roles as both parasites and decomposers (Panzer et al., 2015).

Notably, at the genus taxonomic level, fungal communities exhibit significant variation across different hot springs. Tangchi hot spring is dominated by *Rhizophydium, Aureobasidium, Rhodotorula, Sclerotinia*, and *Tausonia*, while Bantang hot spring exhibits a notable abundance of *Phoma, Coniosporium, Trichomerium Aureobasidium*, and *Rhodotorula*. Compared to temperate freshwater systems (e.g., lakes and rivers), both hot springs exhibited lower fungal richness but higher specialization. For example, genera such as *Aureobasidium* and *Rhodotorula*, which are predominant in both hot springs, show limited distribution in non-thermal freshwater ecosystems, where *Penicillium* and *Aspergillus* genera dominate (Novevska et al., 2010). *Rhodotorula* species exhibit a strong ecological preference for thermally stressed aquatic systems, as evidenced by their presence in both the alkaline hot springs of Kenya's Rift Valley soda lakes and the sulfur-rich geothermal springs of Saudi Arabia (Salano et al., 2017; Selim et al., 2016). Their distribution extends beyond extreme environments to subtropical freshwater ponds (Sutcliffe et al., 2018), indicating a niche preference for thermally influenced (warmer) aquatic habitats. The *Aureobasidium* genus can utilize various waste products and synthesize natural products such as pullulan, fructooligosaccharides, and melanin (Wang et al., 2022).

In Tangchi hot spring, there exists a certain amount of total organic carbon (TOC) and dissolved organic carbon (DOC). *Rhizophydium*, a member of Chytridiomycota, is present with an abundance of 12.36%, which is significantly higher than that in conventional freshwater environments such as eutrophic lakes (7.3%, Wagner et al., 2023) and oligotrophic to mesotrophic lakes (4.2%−5.8%, Paterson, 1963). This species, with its well-known saprotrophic nature, plays a crucial role in the carbon cycle within the hot spring. It can secrete extracellular enzymes like cellulases and chitinases to break down complex organic polymers such as cellulose and chitin from plant debris in the hot spring environment (Grossart et al., 2019). These complex organic polymers are part of the total organic carbon in the hot spring. Through the decomposition process, the large-molecular-weight organic carbon is broken down into smaller molecules, some of which may dissolve into the water, contributing to the dissolved organic carbon pool. This process is essential for the recycling of carbon and nutrients in the ecosystem. The breakdown of organic polymers not only releases carbon back into the environment but also makes nutrients more accessible for other organisms in the hot spring. Moreover, it has been found to promote juvenile fish growth and improve water quality (Lai et al., 2021). The presence and activity of *Rhizophydium* help maintain a balanced carbon and nutrient cycle in the hot spring, which is closely related to the levels and forms of total organic carbon and dissolved organic carbon, ultimately influencing the overall ecological health of Tangchi hot spring.

Bantang hot spring has higher levels of calcium, magnesium, and sulfate ions, along with a relatively high total organic carbon content (21.5 mg/L). *Phoma* and *Coniosporium* were identified as dominant genera in the Bantang hot spring, but absent in the Tangchi hot spring, indicating that they prefer acidic environments. *Phoma* has also been found in Sambe hot springs (slightly acidic and approaching neutral) in Japan, and it is related to manganese metabolism (Sasaki et al., 2013). Moreover, it is reported that *Phoma* has an emulsifying effect on diesel (Lima et al., 2016). *Phoma* has been found not only in hot springs but also in other freshwater environments, such as the Xijiang River in China (Liu et al., 2021), and the acidic Tinto River in southwestern Spain (Lopez-Archilla et al., 2004). Literature indicates that *Coniosporium* is rarely observed in aquatic environments but is commonly reported in terrestrial niches such as soil (Yang et al., 2023), rock surfaces (De Leo et al., 1999), and plant organs (Li et al., 2018). The high-temperature environment of Tangchi hot spring requires fungi to possess thermostable enzyme systems. For example, proteases and amylases in *Saccharomyces* can retain catalytic activity at high temperatures, enabling it to carry out carbohydrate and protein metabolism (Rao et al., 1998).

This study shows that both Tangchi hot spring and Bantang hot spring boast extremely rich and diverse fungal species resources. Moreover, due to the different environmental conditions of the two hot springs, including factors such as temperature, pH, and hydrochemical parameters, these differences have led to significant disparities in the fungal community structures between the two hot springs. This variability reflects the importance of environmental conditions in shaping ecosystems and also provides a scientific basis for the protection and management of these unique natural environments. However, at present, it is impossible to accurately analyze how environmental factors affect the fungal community structure in these two hot springs. Interestingly, the high proportion of unknown fungi (5.13%−9.52%) suggests the presence of novel lineages adapted to geothermal niches, a finding not commonly reported in most freshwater studies. This indicates a certain degree of novelty in the fungi of the two hot springs.

## 5 Conclusion

In this paper, high-throughput sequencing technology was employed for the first time to examine the fungal diversity of Tangchi and Bantang hot springs in Hefei. The findings revealed that the two hot springs possess abundant fungal resources. The richness and evenness of fungi in Tangchi hot spring are greater than those in Bantang hot spring.

The differences in environmental conditions between Tangchi and Bantang hot springs have led to distinct fungal community structures. Fungi in these environments adapt to different temperature, pH, and substrate conditions through various survival strategies and play crucial roles in organic matter decomposition, biogeochemical cycling, and microbial interactions.

Future research can further integrate multi-omics technologies such as metagenomics, metatranscriptomics, and metabolomics to deeply uncover the interaction mechanisms between fungi and the environment, as well as the specific functions and contributions of fungi in hot spring ecosystems. Meanwhile, studying the interactions between fungi and other microorganisms (such as bacteria and archaea) is of great significance for comprehensively understanding the structure and function of hot spring ecosystems.

## Data Availability

The original contributions presented in the study are publicly available. This data can be found here: https://www.ncbi.nlm.nih.gov, accession numbers SRR20831103, SRR20831104, PV773522-PV773934, PV746814-PV747184.
